# DRESS and Stevens–Johnson Syndrome Overlap Secondary to Allopurinol in a 50-Year-Old Man—A Diagnostic and Treatment Challenge: Case Report

**DOI:** 10.3390/life13122251

**Published:** 2023-11-24

**Authors:** José Dario Martínez, Rodolfo Franco, Luis Manuel Sáenz, Americo Guadalupe Alvarado, José Antonio García, Sergio Máximo Delgado, Marius-Anton Ionescu, Camelia Busilă, Alin Laurentiu Tatu

**Affiliations:** 1 Department of Internal Medicine, Faculty of Medicine, Hospital Universitario José Eleuterio González, University Autonomous of Nuevo León, Monterrey 66455, Mexico; americo.alvaradova@uanl.edu.mx; 2 Department of Human Pathology, Faculty of Medicine, Hospital Universitario José Eleuterio González, University Autonomous of Nuevo León, Monterrey 66455, Mexico; rodolfo.francomrqz@uanl.edu.mx (R.F.); antonio.garciamnz@uanl.edu.mx (J.A.G.); 3 Faculty of Medicine, University Hospital José Eleuterio González, University Autonomous of Nuevo León, Monterrey 66455, Mexico; luis.saenzmdn@uanl.edu.mx (L.M.S.); sergio.delgadoj@uanl.edu.mx (S.M.D.); 4 Department of Dermatology, University Hospital Saint Louis, 63110 Paris, France; dr.toni.ionescu@gmail.com; 5 Clinical Medical Department, Faculty of Medicine and Pharmacy, “Dunarea de Jos” University, 800008 Galati, Romania; dralin_tatu@yahoo.com; 6 Dermatology Department, “Sfanta Cuvioasa Paraschiva” Hospital of Infectious Diseases, 800179 Galati, Romania

**Keywords:** allopurinol, eosinophilia, chronic kidney disease, DRESS syndrome, SJS

## Abstract

Drug reaction with eosinophilia and systemic symptoms (DRESS) syndrome is a drug reaction commonly related to eosinophilia, from uncertain epidemiology, and without consensus for diagnosis and treatment globally. It presents a great challenge in its management and is characterized by fever, lymphadenopathy, skin rash, and multisystemic involvement. An aggressive and difficult-to-manage clinical case is presented in a 50-year-old man with chronic kidney disease due to diabetes mellitus type 2 and systemic arterial hypertension, who developed an unusual variant similar to DRESS and Stevens–Johnson syndrome (SJS) overlap secondary to allopurinol, with skin manifestations without eosinophilia, but fulfilling clinical and laboratory criteria for DRESS and SJS syndrome.

## 1. Introduction

DRESS syndrome is a rare but potentially life-threatening hypersensitivity reaction to drugs that can involve multiple organ systems. It was first described in 1996 by Bouquet, and it belongs to the group of severe adverse reactions to drugs (SCARs). The other SCARs are acute generalized exanthematous pustulosis (AGEP), Stevens-Johnson syndrome (SJS), and toxic epidermal necrolysis (TEN) [1].

The incidence of DRESS syndrome is estimated to be 1 in 1000–10,000 drug exposures. It is more common in certain populations, particularly African-American and Caribbean populations [2]. There is no gender preference, and the age of onset can range from infancy to adulthood. The most associated drugs with DRESS syndrome are aromatic anticonvulsants such as phenytoin, carbamazepine, and phenobarbital, as well as sulfonamides, dapsone, minocycline, allopurinol, calcium channel blockers, terbinafine, nonsteroidal anti-inflammatory drugs, and antiretroviral drugs [1].

One of the main clinical manifestations of DRESS syndrome is a maculopapular rash, which can be accompanied by fever and other systemic symptoms [3]. The onset of symptoms usually occurs within 2 to 6 weeks after exposure to the culprit drug. Fulminant liver failure is the most common cause of death, with a mortality rate of 10–20%. The true incidence of DRESS/DIHS is still unknown, but it has been reported at around 10 per million. DRESS syndrome can recur; a systematic review that included 42 articles concluded that the average time to recurrence is 124 days, it usually presents with a higher fever and a higher number of eosinophils, generally attributed to another causal drug. In this study, it is mentioned that the survival of recurrences was 71% [4,5,6].

SJS is a rare, life-threatening mucocutaneous reaction that can also be caused by drug exposure. It has an incidence of 1.5–6.0 cases per million, and it is characterized by the detachment of the epidermis from the dermis, leading to widespread skin blistering and mucosal erosions. SJS is differentiated from TEN by the percentage of body surface area affected: <than 10% for SJS, 10–30% for SJS/TEN overlap, and >30% for TEN [7].

A study conducted in 2023 aimed to estimate the incidence of SJS/TEN and included a total of 51,040 hospitalizations. The results showed a predominance of SJS (73%), followed by the SJS/TEN overlap (15.3%) and TEN (14%). Females were more frequently affected (57.2%), and the highest mortality was observed in patients with TEN (15%). Advanced age, chronic kidney disease, pneumonia, and neoplasms were also associated with mortality in SJS/TEN [8].

Although there are no specific literature or clinical studies available on the overlap of DRESS/SJS syndrome, these SCARs share overlapping clinical manifestations that can sometimes make it challenging to differentiate them. The diagnostic and therapeutic approach to treating DRESS/SJS overlap is a complex issue that requires further research.

## 2. Case Report

**Figure 1:**
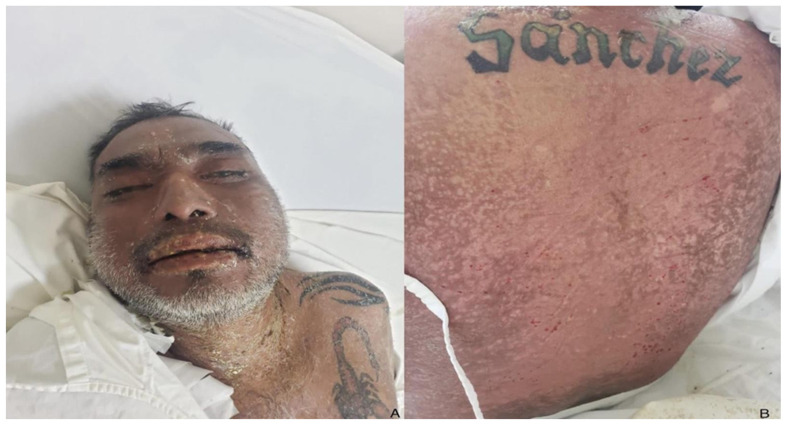
Clinical findings show facial edema with scaling and involvement of the oral and conjunctival mucosa (**A**). Back with scaling erythematous exanthema and positive Nikolsky sign (**B**).

**Figure 2:**
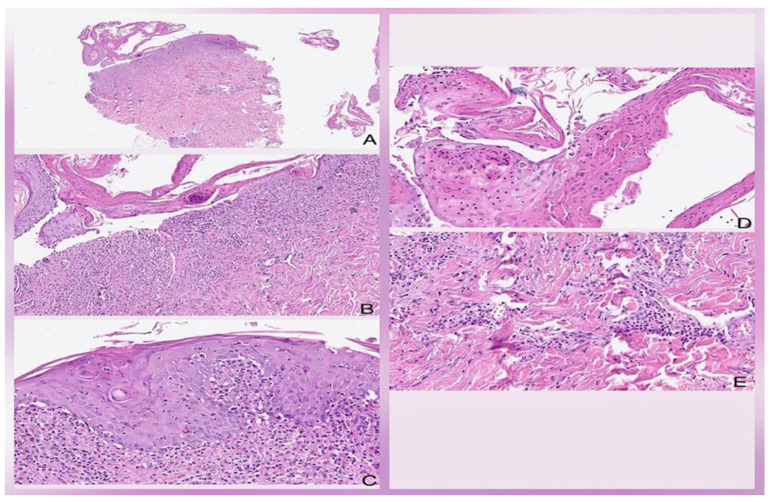
Histologic findings are characterized by the presence of spongiotic and acantholytic lesions, with numerous full-thickness apoptotic and necrotic keratinocytes of the epidermis in some areas (**A**). Dense inflammatory infiltrate of the mixed interface, predominantly lymphocytic and polymorphonuclear (**B**). Disruption of the epidermis with numerous apoptotic bodies, incontinence of pigment, and extravasation of erythrocytes (**C**). Acantholysis and full-thickness necrosis of the epidermis (**D**). Vasculitis at the level of deep vessels is associated with a dense lymphocytic and neutrophilic infiltrate (**E**). Hematoxylin and eosin. Original magnification 2× (**A**), 10× (**B**), 20× (**C**–**E**).

A 50-year-old man arrived at the emergency department with a widespread scaly rash covering his face, chest, abdomen, and back. He also had facial swelling, lymphadenopathy, and mucous membrane involvement (see Figure 1A,B). Upon admission, his physical examination revealed a temperature of 37.8 °C, weight of 80 kg, height of 1.78 m, and a BMI of 25.3. According to family members, he had been taking 300 mg of allopurinol for two weeks before the rash’s appearance as he was diagnosed with hyperuricemia. He had also experienced a fever and upper respiratory tract illness the week before. The patient had a history of type 2 diabetes mellitus for 15 years, multiple lower limb amputations due to diabetic neuropathy and poorly controlled hypertension being treated with losartan and metformin and rapid-acting insulin. Upon admission, we stopped the suspected drug using the Naranjo score. The patient was uncooperative and obtunded. Laboratory abnormalities included a Hb of 8.97 g/dL, hematocrit (HTO) of 29.3%, white blood cells (WBC) of 19.3 K/µL, eosinophils of 0.002 K/µL, platelets of 295,000/mm^3^ plasma glucose level of 143 mg/dL, serum creatinine level of 10.3 mg/dL, BUN level of 150 mg/dL, serum potassium level of 7.2 mmol/L, serum sodium level of 156 mmol/L, and abnormal liver function tests including AST level of 120 UI/L, ALT level of 113 UI/L, ALP level of 439 UI/L, total bilirubin level of 3.3 mg/dL, direct bilirubin level of 1.9 mg/dL, and LDH level of 331 UI/L. We consulted nephrology for hemodialysis and dermatology for a suspected drug-induced skin reaction. Dermatology performed a skin biopsy, which revealed a spongiotic and acantholytic lesion, suggesting an epidermal necrolysis (see Figure 2A–E). Although no tests were carried out to identify the reactivation of HHV 6–7 or EBV, the human pathology department diagnosed Stevens–Johnson syndrome, and the clinical features met the criteria for DRESS. We initiated intravenous corticosteroid therapy at a dose of 4 mg/kg/BID, which was later increased to 8 mg/kg/BID due to poor clinical response (see Figure 1B).

The patient met 6 out of 7 RegiSCAR criteria for DRESS and showed clinical features of SJS. The DIHS severity score was 8. Despite intensive multidisciplinary management, the patient’s renal damage and direct toxicity of allopurinol resulted in the need for oxygen and respiratory distress, followed by cardiac arrest and death on the ninth day of hospitalization.

## 3. Discussion

The pathophysiological basis of the disease is not fully understood, but it is identified as a type 4b delayed hypersensitivity mechanism according to the Coombs and Gell classification [9]. There are also different proposed mechanisms, including issues in drug metabolism, slow acetylation, and reactivation of the Epstein Barr, HSV 6, and 7 viruses [10]. Recent evidence indicates an association between specific HLA haplotypes and the use of certain medications with a higher risk of developing this syndrome. For instance, HLA B 13:01 haplotype has been linked to the use of dapsone, HLA B 58:01 to allopurinol, and HLA B 32:01 to vancomycin [11]. DRESS syndrome is characterized by cutaneous and hematological manifestations with multisystemic involvement. Symptoms usually start between 2 and 90 days with an average of 23 days after consumption of the causative drug [12]. It usually involves a prodromal period characterized by fever, lymphadenopathy, pruritus, and flu-like symptoms [9].

In a recent study that included 125 patients who met DRESS criteria, it was found that the rash usually involves typically >50% of the body surface area (BSA) in 97.6% of cases a maculopapular morphology shown to be the most prevalent (84.6%). Typical facial edema appears in up to 53.8% and mucosal involvement occurs in up to 32.8% [5]. In another study that included 27 patients with DRESS, 100% of the sample had a history of fever, 88.8% developed lymphadenopathy, 92.5% had eosinophilia, and the liver was primarily involved in 100% of cases [13]. The liver plays a central role in this disease and is the main cause of death. A systematic review, aimed at identifying other affected organs, indicated that esophagitis, gastritis, enteritis, colitis, pancreatitis, and even fulminant type 1 diabetes mellitus and type 2 diabetes mellitus may also be present in this syndrome [14]. The Naranjo score is a questionnaire that includes 10 questions whose objective is to determine the presence of an adverse drug reaction. A score of 0 suggests a doubtful diagnosis, 1–4 points indicate a possible reaction, 5–8 points suggest probability, and a score ≥9 points establishes the diagnosis of an adverse drug reaction. Naranjo score alone has low sensitivity, and clinical judgment is necessary to avoid underestimation [15,16]. Several diagnostic criteria for DRESS syndrome exist, including those proposed by Bouquet in 1996, criteria by the RegiSCAR group, and those established by the Japanese J-SCAR consensus. (Table 1).

Drugs associated with DRESS syndrome are associated with specific organ damage, allopurinol has been shown to target the kidney, phenytoin causes liver damage, and carbamazepine causes damage to both. (Table 2).

A skin biopsy is necessary to rule out other potential conditions, as the observed changes are not usually pathognomonic of DRESS. These changes include spongiosis, subcorneal pustules, apoptotic keratinocytes, mixed infiltrates of the superficial dermis, vacuolar changes, and extravasation [17]. The differential diagnosis should include other severe cutaneous adverse reactions (SCARs) such as SJS, TEN, and SJS-TEN overlap. The latter conditions are often related to the same drugs linked to DRESS syndrome and share a similar prodromal period. SJS/TEN may manifest in the skin as erythematous or violaceous patches, atypical targetoid lesions, bullae, erosions, and necrosis. The bullae is usually associated with a positive Nikolsky sign. Around 80% of patients with SJS/TEN may have mucosal involvement [7]. Another SCAR to consider in the differential diagnosis is AGEP, which manifests as non-follicular pustules on an erythematous base, distributed favorably to the extremities (44.2%), trunk (23.3%), face (23.3%), and potential involvement the oral mucosa (11.6%) [18]. In the current context, the differential diagnosis should include adverse skin reactions induced by the vaccine vs. COVID-19, which can cause redness to morbilliform to urticarial rashes [19].

Studies have also identified various factors associated with mortality in patients with SJS/TEN, including advanced age, chronic kidney disease, pneumonia, and neoplasms [8]. Additionally, research has demonstrated that the number of criteria fulfilled according to the RegiSCAR group’s classification system can indicate the aggressiveness of DRESS, with facial edema being a particularly significant cutaneous parameter in determining a more severe form of the syndrome [20]. Another study involving 91 patients diagnosed with DRESS reported 13 fatalities, with specific clinical markers such as age over 40, increased heart rate, fever, elevated BUN levels, leukopenia or leukocytosis, and elevated ALT and AST levels associated with poor prognosis [21]. The severity scoring system for DiHS/DRESS has proven useful in monitoring severity, predicting prognosis, and stratifying the risk of developing CMV disease and complications [22]. The initial step in treatment is the immediate discontinuation of the offending drug [1]. The primary pharmacological treatment for DRESS involves systemic corticosteroids. Recommended doses include intravenous dexamethasone (4–8 mg/kg/every 8–12 h) and IV methylprednisolone (10–30 mg/kg/dose) during the first week, followed by tapering with prednisolone (0.5–2 mg/kg/day) administered in 2–3 months. Systemic corticosteroid treatment is gradually tapered over 3 months [23]. As an alternative treatment for DRESS, the effectiveness of cyclosporine, a calcineurin inhibitor and potent immunosuppressant has been demonstrated. This is particularly beneficial for patients who experience adverse reactions to corticosteroids. The initial dose of cyclosporine is 3–5 mg/kg/day, the dose is increased or decreased up to 50 mg based on the response of the patient, and the duration of treatment ranges from 35 to 76 days [24].

A retrospective cohort study published in 2023 whose objective was to compare the use of systemic steroids vs. topical use in the treatment of DRESS syndrome included 94 patients, 44% received treatment with topical steroids, while the remaining 56% received IV steroids. Patients treated with IV corticosteroids had a higher risk of infectious complications; however, no difference in mortality or hospital stay was found. The study concludes that the use of topical corticosteroids is controversial, but may be a safe and effective alternative, but with a slow response in mild-moderate DRESS [25]. Treatment with IV steroids should not be changed rapidly to topical therapy; instead should be tapered progressively in order to prevent relapses, even when the clinical evolution is favorable [26].

## 4. Conclusions

The case of our patient presented a diagnostic challenge, showcasing a rare and potentially lethal combination of DRESS and SJS syndromes. Despite the absence of eosinophilia, which is characteristic of DRESS, the patient fulfilled the majority of the diagnostic criteria established by both the RegiSCAR and J-SCAR groups. Additionally, the patient exhibited a scaly exanthema, a feature observed in a small percentage of patients with DRESS [3,13]. Notably, our patient had severe mucosal and eye involvement, similar to SJS patients, and displayed a positive Nikolsky sign in certain areas of his body.

Managing this case was further complicated by the patient’s chronic kidney disease, exacerbated by allopurinol-induced kidney damage. This complexity made it challenging to adequately replenish insensible losses and excluded the use of cyclosporine, a drug that has shown utility in treating DRESS/SJS. Certain clinical and biochemical parameters, including elevated thymus and activation-regulated chemokine (TARC) levels, C-reactive protein levels over 5 mg/L, extensive skin rash covering more than 35% of the body surface area, and eosinophilia over 6%, have been identified as predictors of severe DRESS [27].

It should be emphasized that true overlaps, where a patient meets the diagnostic criteria for two SCARs simultaneously, are very uncommon but can occur. The limited scientific literature on overlaps is surprising, considering that the causative drugs are typically the same. Reported cases, such as the overlap between DRESS and SJS due to allopurinol and DRESS and AGEP due to piperacillin/tazobactam, highlight the importance of suspecting overlaps and acting swiftly when overlapping clinical or histopathological features are present [28,29]. A retrospective study aimed at identifying overlaps among SCARs revealed that 21% of the 216 patients studied had two possible diagnoses, with only 2.1% being true SCARs [30]. Additionally, a series of two cases of DRESS/SJS-TEN overlap due to benznidazole reported by Gonzalez Ramos et al. emphasizes the diagnostic challenge of this situation and the need for quick suspicion and treatment due to its potential life-threatening nature [31].

In conclusion, our patient features clinical, laboratory, and biopsy parameters that meet the criteria for DREES and SJS. It was a rare and complex case in the diagnosis and management that requires high suspicion and rapid intervention because of its potential lethality. Both drug reactions are by themselves life-threatening conditions and together are even worse. Further research and understanding of SCAR´s overlaps are necessary to improve diagnostic accuracy and guide clinicians in managing these complex cases.

## Figures and Tables

**Table 1:** Diagnosis criteria for DRESS [1].

RegiSCAR	J-SCAR	Bouquet
Hospitalization	A maculopapular rash developed more than 3 weeks after starting treatment with any drug.	Drug-related skin rash
Acute skin rash	Persistent clinical symptoms after discontinuation of the offending drug.	Hematological abnormalities. Atypical lympho cytes.
		Systemic envelope
Suspicion of reaction caused by drugs Fever >38 °C Growing of lymph nodes > 2 places Hematological abnormalities (Lymphocyto sis or lymphocytopenia, eosinophilia, thrombocytopenia)	Fever > 38 °C. Hepatic abnormalities. Leukocytosis. Atypical lymphocytes. Lymphadenopathy. HSV 6/7 reactivation.	Lymphadenopathy >2 cm Hepatitis with elevated transaminases >2 times normal Interstitial nephritis
		Interstitial pneumonitis

**Table 2:** Drugs and target organs [1,5].

Drug	Target Organ
Allopurinol	Kidney
Carbamazepine	Heart Liver and kidney
Ampicillin Dapsone Minocycline	Liver, lung and heart
Phenytoin	Liver

## Data Availability

The data included in this manuscript cannot be shared publicly, due to the need to protect the privacy of the included subjects. Data may be shared upon reasonable request to the corresponding author.

## Abbreviations

DRESS	Drug reaction with eosinophilia and systemic symptoms
SCARs	Severe cutaneous adverse reactions
SJS	Stevens–Johnson Syndrome
TEN	Toxic epidermal necrolysis
AGEP	Acute generalized exanthematous pustulosis
HLA	Human leukocyte antigens
DIHS	Drug-Induced Hypersensitivity Syndrome
HSV	Herpes Simplex Virus
TARC	Thymus and activation-regulated chemokine
